# Diagnostic and prognostic biomarkers in heart failure and myeloproliferative neoplasms: clinical applications and risk scores – a narrative review

**DOI:** 10.1186/s40959-026-00518-7

**Published:** 2026-06-06

**Authors:** Ionela-Raluca Muraretu, Adriana Luminita Gurghean, Adrian Gradinaru, Mircea Bajdechi, Ilinca Savulescu-Fiedler, Anca-Maria Mihailescu, Adriana Mihaela Iliesiu

**Affiliations:** 1 “Carol Davila” University of Medicine and Pharmacy, Bucharest, 050474 Romania; 2 Department of Internal Medicine and Cardiology, Coltea Clinical Hospital, Bucharest, 030167 Romania; 3 Faculty of Medicine, “Ovidius” University of Constanta, Constanta, 900470 Romania; 4 Department of Cardiology, “Prof. Dr. Theodor Burghele” Clinical Hospital, Bucharest, 050659 Romania; 5 Bucharest, Romania

**Keywords:** Heart failure, Myeloproliferative neoplasms, Biomarkers, Risk stratification, Risk score, Cardio-oncology

## Abstract

Heart failure (HF) remains a leading cause of morbidity and mortality worldwide and frequently coexists with Philadelphia-negative myeloproliferative neoplasms (MPNs), which share thrombo-inflammatory pathways that increase cardiovascular risk. HF is often underestimated in this population, and current risk models rarely include variables specific to this complex cardio-hematologic setting. From a cardio-oncology perspective, MPNs represent a prototypical at-risk cancer population in whom chronic inflammation, clonal hematopoiesis, and cancer therapy converge to accelerate cardiovascular damage.

This narrative review summarizes current evidence on diagnostic and prognostic biomarkers, imaging parameters, and validated HF and MPNs risk scores, and proposes a pragmatic hybrid framework for integrated HF-MPNs cardiovascular risk assessment. PubMed, Embase, and Web of Science were searched up to December 2025 using combinations of terms related to HF, MPNs, biomarkers, imaging, and risk models. In HF, natriuretic peptides and cardiac troponins remain central for diagnosis and short-term prognosis, whereas emerging biomarkers such as galectin-3, soluble ST2, and GDF-15 refine long-term risk prediction. Advanced imaging tools, including global longitudinal strain, left atrial strain, and cardiac magnetic resonance tissue characterization, provide prognostic value beyond the left ventricular ejection fraction. Multidimensional HF models (e.g., MAGGIC, SHFM, BCN Bio-HF, H2FPEF, 3A3B, GWTG-HF, ADHERE, and EHMRG) combine different variables to estimate mortality and hospitalization risk. In MPNs, blood counts, driver mutations (JAK2, CALR, MPL), inflammatory markers, and scores such as IPSET-thrombosis, DIPSS/DIPSS-plus, and MIPSS stratify thrombotic risk, survival, and leukemic transformation but seldom incorporate HF-specific variables.

No single biomarker or score fully captures the bidirectional cardiovascular risk in patients with HF and coexisting MPNs. We propose a simple 0–8-point composite algorithm that combines key HF and MPNs variables (natriuretic peptides or troponins, HF risk scores, driver mutations, leukocytosis/thrombocytosis, LDH) to identify high-risk HF–MPNs phenotypes across ACC/AHA HF Stages A–C, suitable for structured cardio-oncology follow-up. Prospective validation of this hybrid algorithm in dedicated cardio-oncology cohorts and the development of MPNs-adapted HF guidelines are key future priorities.

## Introduction

Heart failure (HF) is a major public health problem, affecting more than 64 million people worldwide, conferring high mortality, recurrent hospitalizations, impaired quality of life, and high healthcare costs [1, 2]. Philadelphia-negative myeloproliferative neoplasms (MPNs), represented by polycythemia vera (PV), essential thrombocythemia (ET), and primary myelofibrosis (PMF), are clonal hematologic malignancies characterized by excessive thrombotic burden and chronic inflammation that increase cardiovascular risk [3, 4]. Cardiovascular manifestations in MPNs extend beyond thrombosis to progressive HF, coronary artery disease, systemic or pulmonary hypertension (PH), valvular heart disease, and pericardial abnormalities [5, 6]. HF prevalence among patients with hematologic malignancies is around 14% [7].

Although HF and MPNs primarily involve different organ systems, they share key pathophysiological pathways, including chronic inflammation, oxidative stress, endothelial dysfunction, metabolic disturbances, high-output states, and clonal hematopoiesis [3, 8]. Epidemiological data suggest that MPNs confer approximately a two-fold higher incidence of HF compared with non-MPNs controls, while HF itself may increase incident cancer risk, highlighting a bidirectional HF-cancer relationship [9, 10]. Together, these data support the concept of a vulnerable cardio-hematologic phenotype, justify considering MPNs as at least a Stage A HF condition, and highlight the need for systematic cardiovascular monitoring [8, 11, 12].

This narrative review synthesizes evidence on diagnostic and prognostic biomarkers, imaging, and validated risk scores in patients with HF and MPNs. The aim is to propose a pragmatic framework for integrated HF-MPNs assessment, identifying evidence gaps and future research priorities. This review is positioned at the intersection of cardiology, hematology and cardio-oncology, with a specific focus on how HF risk in MPNs patients can be systematically assessed and monitored.

### Search strategy

This narrative review is based on a structured literature search of PubMed, Embase, and Web of Science, performed up to December 2025. Combinations of keywords related to HF, MPNs, biomarkers, imaging, and risk scores were used. We primarily included clinical studies and review articles reporting the diagnostic or prognostic value of biomarkers, imaging parameters, or multivariable risk models for cardiovascular outcomes, thrombotic events, or mortality in HF and/or MPNs. No formal quality scoring or meta-analysis was performed; evidence was synthesized narratively.

## Heart failure in patients with myeloproliferative neoplasms: diagnostic and prognostic frameworks

HF is a complex clinical syndrome driven by neuroendocrine activation, inflammation, and fibrosis, diagnosed through clinical history, physical examination, symptoms, imaging, and biomarkers. HF is classified by left ventricular ejection fraction (LVEF) into reduced (HFrEF, LVEF ≤ 40%), mildly reduced (HFmrEF, LVEF 41–49%), and preserved (HFpEF, LVEF ≥ 50%) phenotypes [13].

Diagnosis of MPNs follows WHO 2022 criteria, combining peripheral blood abnormalities, bone marrow morphology, and driver mutations (JAK2 V617F, CALR, and MPL) alongside high-molecular-risk variants (ASXL1, EZH2, and TP53) [14]. Table 1 summarizes diagnostic features and driver mutations characterizing each classical MPNs subtype.

**Table 1:** Clinical, hematologic and molecular features of classical MPNs – adapted from [15]

Disease	Clinical manifestation	Laboratory findings	Driver mutations (approximate frequency)
PV	Thrombotic events, neurological symptoms, pruritus, plethora, splenomegaly	Elevated hematocrit (> 52% in men, > 48% in women); bone marrow panmyelosis	JAK2 V617F or exon 12 (> 95%)
ET	Thrombosis, microvascular disturbances, bleeding tendency	Plt > 450 × 10^9^/L, normal hemoglobin and CRP, megakaryocytic hyperplasia and atypia	JAK2 (55%), CALR (25%), MPL (5%)
PMF	Splenomegaly, extramedullary hematopoiesis, constitutional symptoms (fatigue, weight loss, night sweats)	Teardrop-shaped erythrocytes, bone marrow fibrosis (grade ≥ 1), variable cytopenias	JAK2 (55%), CALR (30%), MPL (5%)

*Abbreviations*: *CRP* C-reactive protein, *Plt* platelets

### Clinical parameters, comorbidities and clinical indicators

Traditional clinical parameters guide HF risk assessment in MPNs patients. Older age and advanced New York Heart Association (NYHA) functional class are consistent predictors of mortality and rehospitalization [16]. Lower systolic blood pressure signals poorer survival through multifactorial mechanisms, including reduced cardiac output, advanced disease stage, neurohormonal dysregulation, comorbidities, and treatment effects [17–19]. MPNs-specific modifiers include age over 60 years, often linked to higher JAK2 V617F allele burden and increased thrombotic and leukemic risk, as well as prior thrombosis, the main predictor of recurrence, independent of mutations or cardiovascular risk factors [20–22].

HF etiologies in MPNs are heterogeneous, each conferring a distinct and often additive risk profile. Ischemic heart disease (50% of all HFrEF) is associated with higher mortality and HF hospitalization rates than non-ischemic etiologies. Recognizing the dominant HF mechanism (ischemic, high-output, valvular, or inflammatory) is therefore critical for individualized cardio-oncology management [8, 23, 24].

Shared comorbidities further modulate prognosis. Atrial fibrillation (AF), renal or hepatic dysfunction, diabetes mellitus, and anemia are powerful predictors of adverse outcomes in HF and significantly amplify cardiovascular and thrombotic risk in MPNs [25–27]. Anemia reduces exercise capacity, increases hospitalization and mortality in HF, but also identifies transfusion-dependent high-risk PMF. Blood transfusions create a therapeutic paradox: essential for MPNs cytopenias but predisposing to volume overload, HF decompensation, and leukemic transformation [28–30].

Spleen and liver stiffness correlate with bone marrow fibrosis degree, with spleen stiffness notably greater in PMF and PV than in ET. Serial evaluation of bone marrow fibrosis and spleen size in patients receiving JAK1/2 inhibitors can refine risk evaluation and treatment planning [3, 14, 31].

### Biomarkers at the HF-MPNs interface

Biological markers provide mechanistic and prognostic insight into both HF (myocardial stress, inflammation, and neurohormonal activation) and MPNs (hematologic and molecular drivers of progression). Current guidelines formally recommend only natriuretic peptides and cardiac troponins for HF [32]. In MPNs, these markers should be interpreted against a background of chronic inflammation, clonal hematopoiesis, and prothrombotic state.

#### Hemodynamic stress and injury markers

Natriuretic peptides (NPs), particularly BNP and NT-proBNP, remain the gold standard markers for HF diagnosis and risk stratification. Low levels rule out stable chronic HF (BNP < 35 pg/mL, NT-proBNP < 125 pg/mL) but require higher cut-offs in acute settings (BNP < 100 pg/mL, NT-proBNP < 300 pg/mL) [13, 33]. Each standard-deviation increase in NT-proBNP is linked to a 1.7-fold rise in major adverse cardiac events [34–36].

High-sensitivity cardiac troponins (hs-cTnT) detect subclinical myocyte injury and are associated with advanced HF, higher risk of (re)hospitalization, and mortality and may also predict new-onset HF after acute myocardial infarction [37, 38]. Admission hs-cTn > 14 ng/L predicts 12-month mortality in acute HF decompensations, while cumulative 12-months exposure (median 150 ng/L/month) offers superior risk stratification (HR 4.14 [95% CI 2.51–6.85]) [39].

In MPNs, NPs and troponins serve as adjunctive indicators of cardiovascular stress and injury. Given the increased burden of thrombosis, HF, and ischemic complications, these markers can uncover subclinical ventricular disfunction or myocardial injury when clinically suspected [8, 40].

#### Inflammation and fibrosis biomarkers

Classical inflammatory markers such as C-reactive protein (CRP), interleukin-6 (IL-6), and tumor necrosis factor-alpha (TNF-α) are key mediators in both HF progression and MPNs pathophysiology, reflecting a shared inflammatory substrate. In HF, elevated CRP reflects systemic inflammation and poor outcomes, IL-6 impairs myocardial function and promotes hypertrophy, and TNF-α drives adverse remodeling and inflammation [41, 42]. In MPNs, elevated cytokines (e.g., IL-1, IL-2, IL-6, IL-8, IL-12, TNF-α, GM-CSF, PDGF, and VEGF) coexist with reduced anti-inflammatory mediators (e.g., IL-4, IL-10), resulting in a pronounced immune imbalance. This chronic inflammatory state drives clonal proliferation, endothelial dysfunction, thrombosis, and potentially cardiac remodeling [42–45]. Distinct cytokine profiles characterize PV, ET, and PMF and correlate with progression, thrombotic risk, and fibrotic transformation [46, 47]. Increased hs-CRP correlates with disease burden and nearly double thrombotic risk in MPNs when exceeding 3 mg/L, whereas higher pentraxin 3 appears protective [48, 49].

Galectin-3 (Gal-3) reflects cardiac inflammation and fibrosis, with levels above 13–17 ng/mL associated with disease severity and adverse outcomes. [50, 51]. In MPNs, Gal-3 overexpression in PV contributes to angiogenesis and modulation of the JAK/STAT pathway, supporting a pathogenic role [52].

Soluble ST2 (sST2) promotes cardiomyocyte apoptosis, hypertrophy, and fibrosis, thereby contributing to HF progression, and levels above 35 ng/mL predict hospitalization and mortality risk [53, 54]. In MPNs, the ST2/IL-33 axis appears biologically relevant but lacks clinical utility as a routine biomarker [55, 56].

Single biomarkers have limited accuracy, but combining NT-proBNP with Gal-3 or sST2 improve diagnosis and risk prediction of adverse outcomes [57]. Gal-3 predicts short-term mortality and rehospitalization, particularly in HFpEF, while sST2 provides prognostic information across HF phenotypes and is associated with reverse remodeling [25, 37, 58, 59].

Growth differentiation factor-15 (GDF-15) is a stress-responsive cytokine, elevated in decompensated HF and independently predicts adverse events, sometimes outperforming NT-proBNP, including in HFpEF [32, 60–62]. In MPNs, GDF-15 is upregulated in PV and ET, particularly in JAK2-V617F–positive patients, but its role remains primarily biologic rather than clinical [63].

Overall, these data support a multi-biomarker approach (NT-proBNP, hs-cTn, Gal-3, sST2, GDF-15, CRP) for HF risk assessment in MPNs, which can be layered onto standard hematologic parameters in cardio-oncology clinics.

#### Hematologic and metabolic biomarkers

Peripheral blood count abnormalities are central to MPNs diagnosis: erythrocytosis and elevated hemoglobin in PV, marked thrombocytosis of ET, and variable cytopenias in PMF [64]. Persistent leukocytosis and thrombocytosis signal poor outcome and increased thrombotic risk [65, 66]. Paradoxically, extreme thrombocytosis (≥ 1000 × 10⁹/L) increases bleeding via acquired von Willebrand syndrome [67, 68].

Simple indices derived from blood counts, including neutrophil-to-lymphocyte ratio (NLR), monocyte-to-lymphocyte ratio (MLR), and systemic immune-inflammation index (SII; platelet count × neutrophil count/lymphocyte count), are markers of systemic inflammation in MPNs and correlate with thrombotic risk, disease severity, and overall survival [69]. In PV, NLR ≥ 5 predicts venous thromboembolism [70]. In HF, NLR and SII are linked to disease severity and all-cause mortality [71, 72].

Metabolic markers also overlap: hypoalbuminemia is common in both HF and PMF and is associated with increased mortality. In HF, low albumin reflects inflammation, malnutrition, hepatorenal dysfunction, and volume overload, correlating directly with elevated CRP and inversely with total cholesterol [73–76]. The C-reactive protein-to-albumin ratio (CAR) is a powerful prognostic indicator of chronic HF [37]. Elevated lactate dehydrogenase (LDH) and uric acid reflect increased cellular turnover, oxidative stress, and endothelial dysfunction across both disease spectra, serving as markers of clonal proliferation and disease burden in MPNs and of disease progression in HF [77–79].

These hematologic and metabolic variables are particularly attractive for hybrid HF–MPNs risk models because they are routinely available and inexpensive, making them suitable for longitudinal cardio-oncology follow-up.

#### Molecular and tumor markers

Somatic driver mutations in JAK2, CALR, and MPL define the MPNs phenotypes [80]. JAK2 V617F is present in approximately 97% of PV and over 50% of ET and PMF cases and confers both diagnostic and prognostic value [81, 82]. Higher allele burden (20–50%) is associated with increased venous thromboembolism risk [83, 84]. In JAK2 V617F-negative PV, rare exon 12 mutations define a subgroup with similar features and potential evolution to secondary myelofibrosis [81, 85]**.** Calreticulin (CALR) mutations, seen in ET and PMF but not PV, are more prevalent in PMF and post-ET myelofibrosis, suggesting a role in fibrotic progression [86–88]. CALR-mutated patients exhibit lower leukocyte counts and hemoglobin, higher platelet, and lower arterial thrombotic risk than JAK2- or MPL-mutated cases [89, 90]. MPL mutations in exon 10 (thrombopoietin receptor) occur in a minority of ET and PMF cases and are associated with lower hemoglobin and higher transfusion dependence [91, 92].

Additional non-driver mutations modulate the effects of driver mutations, influence clonal evolution, and contribute to disease progression [87, 93]. High-molecular-risk variants (ASXL1, EZH2, SRSF2, U2AF1 Q157, and IDH1/IDH2) and a higher overall mutational load, particularly in PMF, correlate with more advanced disease, reduced survival and a higher likelihood of leukemic transformation [94, 95]. Approximately 10% of patients remain “triple-negative” but often present these adverse genotypes [93, 96].

Cancer Antigen-125 (CA-125), traditionally a tumor marker, reflects congestion, inflammation, and mechanical stress. Elevated levels are associated with HF severity and higher risk of hospitalization and mortality, especially when combined with NPs in advanced or right-sided HF [97].

Taken together, driver and high-molecular-risk mutations primarily inform hematologic prognosis but also identify subsets in whom aggressive cardiovascular risk factor control and HF surveillance are particularly warranted.

### Imaging-based predictors

Advanced cardiac imaging, mainly echocardiography and cardiac magnetic resonance (CMR), complement biomarkers by refining HF risk stratification, particularly in HFpEF with multiple comorbidities. LVEF distinguish risk for overall mortality, cardiovascular death, and HF hospitalizations, particularly in HFrEF, but its discriminatory value is attenuated in preserved EF, so additional parameters are often needed [98, 99]. Left atrial volume index (LAVI) and left ventricular mass index (LVMI) are independently associated with adverse outcomes across HF phenotypes, with LAVI > 34 mL/m^2^ reflecting chronic atrial remodeling and LVMI > 115 g/m^2^ for men and > 95 g/m^2^ for women indicating LV hypertrophy [100].

Reduced left ventricular global longitudinal strain (LV-GLS less negative than −16% to −18%), reveals subtle systolic dysfunction despite normal LVEF, correlating with wall stress, fibrosis, and diastolic dysfunction [101, 102]. Strain-based indices and right heart parameters are emerging as strong prognostic markers in HFpEF, with reduced LV-GLS (less negative than −18%), impaired left atrial reservoir strain (PALS < 18–20%), and decreased TAPSE/PASP ratio (< 0.36 mm/mmHg, a marker of ventriculo-arterial uncoupling), independently predicting cardiovascular death and HF hospitalizations [103, 104].

While 2D echocardiography remains first-line, 3D-LVEF improves reproducibility for serial monitoring in cardio-oncology patients, facilitating the detection of small changes in LV function [105].

CMR offers detailed myocardial tissue characterization and is widely used for etiologic assessment and risk stratification in HF [106, 107]. In nonischemic cardiomyopathies, myocardial fibrosis detected by late gadolinium enhancement (LGE) combined with an LVEF ≤ 35% is associated with all-cause and cardiac mortality and predicts sudden cardiac death [108, 109].

Bone marrow evaluation is essential for differentiating pre-fibrotic PMF from ET and masked PV and for confirming progression to myelofibrosis. In PV patients with absolute erythrocytosis (Hb > 18.5 g/dL in men or > 16.5 g/dL in women), JAK2 mutations, and low erythropoietin, biopsy may be omitted [110–112]. Magnetic resonance imaging can quantify bone marrow infiltration in PMF, reflecting fibrosis and cellularity, and monitor therapeutic response to identify patients with hematologic progression requiring intensified cardiac screening [113].

## Risk stratification frameworks

### Prognostic models and risk scores in HF

Risk stratification is central to HF management, and several multidimensional scores predict all-cause mortality, sudden cardiac death, and cardiovascular events (see Table 2).

**Table 2:** Overview of major prognostic risk scores in heart failure patients

Model (references)	HF type	Variables and scoring method	Outcomes
CHRONIC HF			
MAGGIC risk score [114]	Mixed EF	Demographics, LVEF, NYHA class blood pressure renal function, comorbidities, HF therapies	All-cause mortality; cardiovascular/HF hospitalization
SHFM [115]	HFrEF > HFpEF	Demographics, NYHA class, blood pressure, ischemic etiology, laboratory variables, comorbidities, and HF medications/devices	1-, 2-, 3-year survival
BCN Bio-HF risk [116]	HFrEF/HFmrEF > HFpEF	Clinical variables, LVEF, renal function, natriuretic peptides, hs-cTnT, and sST2	1-, 2-, 3-year survival
H2FPEF score [117]	HFpEF	Obesity, AF, age, antihypertensive therapy, diastolic indices (E/e’), and PASP	Probability of HFpEF; HF hospitalization and cardiovascular events
3A3B score [118]	HFpEF	Age, albumin, anemia, BMI, natriuretic peptides, and blood urea nitrogen	5-year all-cause mortality
MEDIA echo score [119]	HFpEF	Echocardiographic indices of pulmonary pressure, right heart function, and LV filling pressure	1-year cardiovascular events (death, HF hospitalization)
ACUTE/DECOMPENSATED HF			
GWTG-HF [120]	Any EF	Demographics, blood pressure, renal function, serum sodium, comorbidities	In-hospital mortality (risk categories from < 1% to > 50%)
ADHERE risk score [121]	Any EF	Blood urea nitrogen, systolic blood pressure, creatinine	In-hospital mortality
EHMRG risk score [122]	Any EF	Demographics, vital signs, renal function, troponin, comorbidities, and ECG changes	7-day and 30-day mortality
MELD-XI [123, 124]	Any EF	Creatinine and bilirubin (no INR)	1-, and 3-year all-cause mortality
IMAGING/ADJUNCTIVE			
CMR (LGE based) score [125]	HFrEF/HFpEF	Presence and extent of myocardial fibrosis on LGE, LVEF, LV volumes and mass	All-cause mortality; sudden cardiac death (higher risk with extensive LGE and reduced LVEF)

*Abbreviations*: *GWTG-HF* Get With The Guidelines-Heart Failure, *LGE* late gadolinium enhancement, *MAGGIC* Meta-Analysis Global Group in Chronic Heart Failure, *MEDIA* M-mode Echocardiography–Derived Index for Assessment, *MELD-XI* The Model for End-Stage Disease eXcluding INR, *PASP* pulmonary artery systolic pressure, *SHFM* Seattle Heart Failure Model, *3A3B* Age, Albumin, Anemia, BMI, BNP, BUN

In acute and in-hospital settings, the Get With The Guidelines-Heart Failure (GWTG-HF), Acute Decompensated Heart Failure National Registry (ADHERE), or Emergency Heart Failure Mortality Risk Grade (EHMRG) scores support short-term risk estimation and triage decision. In chronic HF, tools such as the Seattle Heart Failure Model (SHFM), the Meta-Analysis Global Group in Chronic Heart Failure (MAGGIC) score, and the Barcelona Bio-HF (BCN Bio-HF) risk score provide long-term survival estimation and enable dynamic risk reassessment with simple clinical and laboratory inputs [126]. MAGGIC and SHFM are among the most widely validated tools for long-term outcomes in both HFrEF and HFpEF [114, 115].

More recent models enhance predictive power by incorporating biomarkers (hs-cTn, soluble ST2, NT-proBNP in BCN Bio-HF), echocardiographic parameters (the MEDIA echo score), cardiac magnetic resonance metrics (LGE-based CMR scores), or hemodynamic indices tailored to HFpEF (H2FPEF and 3A3B) [116, 119, 125]. Moreover, the H2FPEF and 3A3B scores are tailored to the HFpEF phenotype, reflecting the growing need for phenotype-specific prognostic tools [117, 118].

In patients with MPNs, these HF scores can be applied as in the general population, but their calibration may be imperfect because they do not account for MPNs-specific thrombotic, inflammatory, and hematologic factors.

### Prognostic models and risk scores in MPNs

Prognostic scoring systems for MPNs mainly target three domains: thrombotic risk, survival, and leukemic transformation, and are widely used to guide cytoreduction, transplant decisions, and intensity of hematologic follow-up (see Tables 3, 4 and 5). These models are powerful for hematologic outcomes but largely ignore HF-specific indices, so they may miss important aspects of cardiovascular risk, particularly in patients with established or incipient HF.

**Table 3:** Risk and prognostic scoring systems for PV

**Axis**	**Risk score** (references)	**Variable**	**Risk stratification**
Thrombosis	**Classic thrombotic risk** [127, 128]	Age > 60 Prior thrombosis	Low: age < 60 and no prior thrombosis High: Age ≥ 60 or prior thrombosis
	**MFPS-PV** [129]	Age ≥ 60 (1 p) CVRF^a^ (1.5 p) HR mutation for thrombosis^b^ (1.5 p) Previous thrombosis (2 p)	Low: 0–1 p Intermediate: 1.5–2.5 p High: ≥ 3 p
Survival	**MIPSS-PV** [130]	Age > 67 (2 p) WBC > 15 × 10^9^/L (1 p); Thrombosis history (1 p) SRSF2 mutation (3 p)	Low: 0–1 p (mOS: 24 years); Intermediate: 2–3 p (mOS: 13.1 years) High: ≥ 4 p (mOS: 3.2 years)
Transformation	**Leukemic/myelodysplastic risk factors** [131]	Age > 65 WBC > 15 × 10^9^/L Abnormal karyotype—ASXL1/SRSF2 mutation	Low: no adverse feature High: age > 65 years, ASXL1/SRSF2 mutations, leukocytosis

*Abbreviations*: *ASXL1* Additional Sex Combs-Like 1, *BCOR/BCORL1* BCL6 Corepressor/BCL6 Corepressor Like 1, *CVRF* cardiovascular risk factors, *DNMT3A* DNA (cytosine-5)-methyltransferase 3 alpha, *HR* high risk, *MFPS-PV* Multiple Factor Prognostic Score for PV, *MIPSS-PV* Mutation-Enhanced International Prognostic Score System for Polycythemia Vera, *mOS* median overall survival, *SRSF2* Serine and Arginine Rich Splicing Factor 2, *WBC* white blood cell

^a^Hypertension, diabetes, hyperlipidemia, obesity, smoking

^b^ASXL1, DNMT3A, BCOR/BCORL1

**Table 4:** Risk and prognostic scoring systems in ET

**Axis**	**Risk score** (references)	**Variable**	**Risk stratification**
Thrombosis	**IPSET-thrombosis** [128]	Prior thrombosis (2 p) Age ≥ 60 (1 p) JAK2 V617F (2 p) CVRF (1 p)	Low: 0–1 p Intermediate: 2 p High: ≥ 3 p
	**Revised IPSET-thrombosis** [22]	Age > 60 Thrombosis history JAK2 V617F	Very low: no feature Low: JAK2V617F only Intermediate: age > 60 years High: thrombosis history or both advanced age and JAK2V617F
Survival	**IPSET** [132]	Age ≥ 60 (2 p) WBC ≥ 11 × 10^9^/L (1 p) Thrombosis history (1 p)	Low: 0 p (mOS: not reached) Intermediate: 1–2 p (mOS: 24.5 years) High: 3–4 p (mOS: 13.8 years)
	**MIPSS-ET** [130]	Age > 60 (4 p) Male sex (1 p) WBC ≥ 11 × 10^9^/L (1 p) SF3B1/SRSF2/TP53/U2AF1 mutation (2 p)	Low: 0–1 p (mOS: 34.4 years) Intermediate: 2–5 p (mOS: 14.1 years) High: ≥ 6 p (mOS: 7.9 years)
Transformation	**Leukemic risk** [133, 134]	Plt ≥ 1000 × 10^9^/L (1 p) Abnormal karyotype (1 p)	Overall incidence: 3% 20-year risk of blastic transformation: Low (0 p): 3%; High (1–2 p): 13%
	**Fibrotic progression** [133, 134]	MPL mutation ANC ≥ 8 × 10^9^/L	12%: absence of both risk factors 49%: ≥ 1 factor

*Abbreviations*: *ANC* absolute neutrophil count, *CVRF* cardiovascular risk factors, *IPSET* International Prognostic Score for Essential Thrombocythemia, *MIPSS-ET* Mutation-Enhanced International Prognostic Score System for Essential Thrombocythemia, *MPL* myeloproliferative leukemia virus oncogene, *mOS* median overall survival, *Plt* platelets, *WBC* white blood cell

**Table 5:** Risk and prognostic scoring systems for MF

**Axis**	**Risk score** (references)	**Variable**	**Risk stratification**
Thrombosis	**Conventional risk** [135]	Age > 60 y, JAK2 mutation, WBC ≥ 15 × 10^9^/L	Low: absence of risk factors High: JAK2 mutation + leukocytosis
Survival	**DIPSS** [66]**/** **DIPSS-plus** [136]	**DIPSS**: Age ≥ 65 (1 p) Hb < 10 g/dL (2 p) WBC ≥ 25 × 10^9^/L (1 p) Blasts ≥ 1% (1 p) Constitutional symptoms (1 p) **DIPSS-plus adds**: Transfusion (1 p) Platelets < 100 × 10^9^/L (1 p) Adverse cytogenetics (1 p)	**DIPSS:** Low: 0 p (mOS: not reached) Intermediate-1: 1–2 p (OS: 14.2 years) Intermediate-2: 3–4 p (mOS: 4 years) High: 5–6 p (mOS: 1.5 years) **DIPSS-plus:** Low: 0 p (mOS: 16.4 years) Intermediate-1: 1 p (OS: 6.5 years) Intermediate-2: 2–3 p (mOS: 2.9 years) High: ≥ 4 p (mOS: 1.3 years)
	**MIPSS70-plus v2.0** [137]	Hb: 9–10.9 (male)/8–9.9 (female) (1 p) Hb < 9 (male)/< 8 (female) (2 p) Blasts ≥ 2% (1 p) Constitutional symptoms (2 p) Absence of CALR type-1 mutations (2 p) 1 HMR mutation (2 p) ≥ 2 HMR mutations (3 p) Unfavorable karyotype (3 p) VHR karyotype (4 p)	Very low: 0 p (mOS: not reached) Low: 1–2 p (mOS: 10.3 years) Intermediate: 3–4 p (mOS:7 years) High: 5–8 p (mOS: 3.5 years) Very high: ≥ 9 (mOS: 1.8 years)
Transformation	**Leukemic risk model** [138]	Age > 70 (1 p) Anemia (1 p) Blasts ≥ 3% (2 p) IDH1 (3 p), SRSF2 (2 p), ASXL1 (1 p)	Low: 0–1 p (LE: 8%); Intermediate: 2–6 p (LE: 17%) High: 7–8 p (LE: 57%)
	**MYSEC-PM** [139]	Age (0.15^a^) Hb < 11 g/dL (2 p) Plt < 150 × 10^9^/L (1 p) Blasts ≥ 3% (2 p) Constitutional symptoms (1 p) CALR-unmutated (2 p)	Final risk^b^ Low (mOS: not reached) Intermediate-1 (mOS: 9.3 years) Intermediate-2 (mOS: 4.4 years) High (mOS: 2 years)

HMR (high molecular risk): Presence of a mutation in any of the following genes: ASXL1, EZH2, SRSF2, U2AF1 Q157, or IDH1/2

Unfavorable karyotype: complex karyotype or one or two abnormalities including + 8, 7/7q −, i(17q), 5/5q −, 12p −, inv (3), or 11q23 rearrangements

VHR (very high-risk) karyotype: single/multiple abnormalities of − 7, i(17q), inv (3)/3q21, 12p −/12p11.2, 11q −/11q23, or other autosomal trisomies not including + 8/+ 9 (e.g., + 21, + 19)

*Abbreviations*: *DIPSS* Dynamic International Prognostic Scoring System, *DIPSS-plus* Dynamic International Prognostic Scoring System plus, *Hb* hemoglobin, *IDH1/2* isocitrate dehydrogenase ½, *IPSS* International Prognostic Scoring System, *IPSS-R* Revised International Prognostic Scoring System, *LE* leukemic events, *MIPSS70* Mutation-Enhanced International Prognostic Scoring System for patients ≤ 70 years, *MIPSS70-plus v2.0* Mutation-Enhanced International Prognostic Scoring System for patients ≤ 70 years plus version 2.0, *mOS* median overall survival, *MYSEC-PM* Myelofibrosis Secondary Prognostic Model, *Plt* platelet count, *U2AF1* U2 small nuclear RNA auxiliary factor 1, *WBC* white blood cell count

^a^Age contributes to the overall score through specific points allocated in the nomogram

^b^The MYSEC-PM nomogram is applied to determine the final risk category

#### Polycythemia vera

In PV, arterial and venous thrombosis are the leading cause of morbidity and mortality [140]. Age above 60 years and history of thrombosis are the strongest predictors of vascular events [141–143]. The Multiple Factor Prognostic Score for PV (MFPS-PV) refines thrombotic risk stratification by incorporating cardiovascular risk factors and high-risk mutations [129]. For survival, Mutation-Enhanced International Prognostic Scoring System for PV (MIPSS-PV) adds molecular variables such as SRSF2, guiding cytoreduction [130, 143]. Leukemic transformation, less common than in PMF, links to age, leukocytosis, abnormal karyotypes, and SRSF2/IDH2 mutations, particularly in patients exposed to alkylating agents or radiophosphorus therapy [131, 144]. The main PV scores and their variables are summarized in Table 3.

#### Essential thrombocythemia

ET generally has the most favorable prognosis among classical MPNs, with median survival approximately 18–20 years, though thrombotic events dominate morbidity and mortality [8, 145]. The International Prognostic Score for Thrombosis in ET (IPSET-Thrombosis) and revised IPSET-Thrombosis (r-IPSET-T) integrate clinical and molecular factors to refine thrombotic risk prediction and guide cytoreduction versus antiplatelet therapy [22, 128]. For survival, International Prognostic Score for ET (IPSET-Survival) and Mutation-Enhanced IPSS for ET (MIPSS-ET) refine risk by integrating inflammatory markers and high-molecular-risk mutations (SF3B1/SRSF2/TP53/U2AF1) [130, 132, 146]. Although leukemic and fibrotic transformation are uncommon in ET over 10–15 years, they are associated with older age, extreme thrombocytosis, anemia, leukocytosis, exposure to alkylating agents and adverse genotypes (e.g., ASXL1, SRSF2, and IDH1/2), with MPL mutations and elevated neutrophil counts predicting fibrotic progression (Table 4) [133, 134, 147, 148].

#### Primary myelofibrosis

PMF is the most aggressive form of classical MPNs, with a median overall survival of 6 years, marked by high symptom burden, risk of leukemic transformation, and complications [8, 149]. Table 5 summarizes some of the most widely used risk scores.

Thrombotic events are less frequent than in PV but the risk increases with older age, JAK2 mutation status, and leukocytosis [135]. Survival prognostication has evolved from purely clinical scores, such as the International Prognostic Scoring System (IPSS) and the Dynamic IPSS (DIPSS)/DIPSS-Plus, which dynamically reassess risk by giving more weight to anemia, thrombocytopenia, transfusion dependency, and adverse cytogenetics [66, 136, 149]. More recently molecularly integrated models such as MIPSS70 and MIPSS70 + v2.0, combine clinical variables with high-molecular-risk mutations and karyotype abnormalities, particularly to guide transplant decisions [150, 151]. Leukemic transformation occurs in 10%−20% at 10-year, with post-transformation survival of 3–4 months [144, 152]. Key predictors include a higher blast percentage, cytopenias, unfavorable cytogenetics, triple-negative status and mutations such as ASXL1, SRSF2, RUNX1, CEBPA, and SH2B3 [30, 94]. The Myelofibrosis Secondary to PV/ET Prognostic Model (MYSEC-PM) is specifically designed for secondary myelofibrosis [139]. Treatment-response scores for ruxolitinib and transplant-specific models, refine prognosis in advanced disease in which optimization of cardiovascular status is crucial [153, 154].

Although these PV, ET and PMF scores were not developed with HF endpoints in mind, high-risk categories often coincide with older age, heavy symptom burden, inflammatory activation, anemia or transfusion dependence and adverse genotypes, all of which may amplify HF risk and therefore represent natural anchors for integrating cardiovascular biomarkers and imaging in our hybrid HF–MPNs framework.

## Clinical implications

### ACC/AHA HF stages and pathophysiological mechanisms in MPNs-HF

The 2022 AHA/ACC/HFSA guidelines define HF as a continuum from Stage A (at risk for HF without structural disease or elevated biomarkers), Stage B (pre-HF: structural disease, elevated filling pressures, or persistently elevated natriuretic peptides/troponins without symptoms), Stage C (symptomatic HF), to Stage D (advanced/refractory HF) [12]. In a large claims database, patients with MPNs had a higher incidence of congestive HF than non-MPNs controls (9.27 vs 3.70 per 1000 person-years; adjusted HR 1.64) [10]. In observational cohorts, patients with MPNs and concomitant HF experience higher in-hospital mortality and 90-day cardiovascular readmission rates than non-MPNs patients, with ET and especially myelofibrosis conferring greater 90-day CV and HF-related readmission risk than PV [155].

Chronic systemic inflammation, oxidative stress, and endothelial dysfunction promote diffuse myocardial fibrosis, diastolic dysfunction, and HFpEF, whereas microvascular dysfunction and accelerated atherosclerosis increase the burden of ischemic HFrEF [45, 156, 157]. High-output states (8% of MPNs cases) driven by anemia, hypervolemia, and splenomegaly impose LV stress, predisposing to eccentric remodeling and HF progression [8, 158]. In addition, pulmonary hypertension related to chronic thromboembolic disease or elevated pulmonary vascular resistance may aggravate right HF [159, 160]. Cancer therapies, including cytoreductive agents and JAK inhibitors, bidirectionally modulate risk: attenuating inflammatory signaling and splenomegaly they may mitigate HF risk, but potential off-target cardiotoxicity, fluid retention, and anemia can precipitate HF decompensation, particularly in patients with pre-existing Stage C disease [8, 161, 162].

Taken together, these data support regarding MPNs as a high-risk Stage A HF condition, as chronic thrombo-inflammatory activation, endothelial dysfunction, oxidative stress, anemia/high-output states, and treatment effects create a persistent substrate for structural and functional myocardial injury, mirroring ACC/AHA Stage A criteria despite absent overt disease.

### Risk stratification and monitoring

Patients with MPNs represent a high-risk cardio-oncology population requiring systematic baseline cardiovascular assessment before initiating cytoreductive therapy or JAK inhibitors. Although MPNs-specific data are limited, information is extrapolated from the ESC 2022 Cardio-Oncology Guidelines, that recommend a comprehensive baseline evaluation, including history, ECG, echocardiography with GLS, natriuretic peptides, and cardiac troponins, for all patients starting potentially cardiotoxic cancer therapies [163]. Baseline echocardiography frequently reveals early diastolic dysfunction even in asymptomatic patients, particularly those with JAK2 V617F mutations. This initial assessment identifies patients who may benefit from preventive guideline-directed medical therapy (GDMT), such as sodium-glucose cotransporter-2 (SGLT2) inhibitors, prior to disease-modifying MPNs treatments [160, 164].

**Fig. 1:**
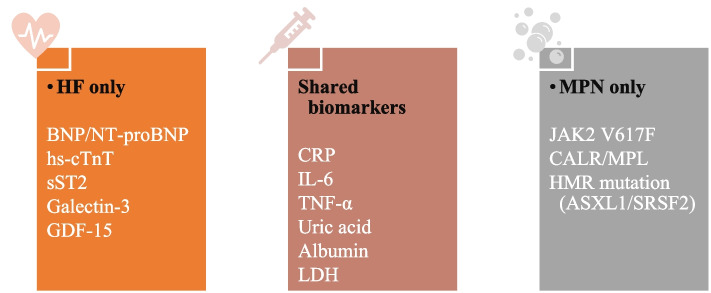
Integrated biomarker profile in patients with HF and MPNs. See abbreviations list

Dynamic risk assessment during JAK inhibitor therapy (every 3–6 months) includes NT-proBNP and hs-cTnT monitoring to detect subclinical cardiotoxicity early. Trends in the levels of inflammatory markers (CRP and IL-6) are correlated with both thrombotic risk and HF decompensation [165, 166]. Figure 1 illustrates the main diagnostic and prognostic biomarkers in HF and MPNs patients, highlighting metabolic–inflammatory overlaps for cardio-oncology follow-up.

Although MPNs-specific echocardiographic GLS data remain limited, frequent subclinical diastolic dysfunction and emerging strain data from high-risk cardio-oncology cohorts support its utility in MPNs patients. GLS surveillance is particularly valuable in preserved LVEF cases, where strain reduction typically precedes systolic dysfunction. In a prospective MPNs cohort with PH-associated HFpEF, right ventricle-GLS < − 19% and LV-GLS identified subclinical dysfunction despite normal LVEF, demonstrating superior mortality prediction (HR 3.2, *p* = 0.003 independent of PASP) versus conventional TAPSE/S′, enabling Stage B detection to prevent HF progression [167].

### Therapeutic decision-making

Optimal HF management in MPNs remains poorly defined. National Comprehensive Cancer Network guidelines promote CV risk factors control but lack HF recommendations [110]. While JAK inhibitors show promise, HF likely arises from shared pathophysiology rather than cardiotoxicity [9, 163].

In the absence of specific HF-MPNs guidelines, the implementation of integrated risk stratification and therapeutic strategies necessitates a cardio-hemato-oncology multidisciplinary team (MDT) approach—as endorsed by the 2022 ESC Cardio-Oncology Guidelines—to optimize GDMT (e.g., SGLT2i ± ARNI), cytoreductive therapies (ruxolitinib/hydroxyurea), and serial monitoring of NT-proBNP/GLS, thereby minimizing HF decompensations in this high-risk population [163, 168].

The clinical challenge is balancing antithrombotic therapy against bleeding risk while optimizing HF management. No formal guidelines exist for managing new-onset or decompensated HF during cytoreductive or JAK-inhibitor therapy in MPNs. The strategies outlined below represent a pragmatic approach extrapolated from trial data and expert consensus.

In patients with MPNs requiring cytoreduction, baseline HF risk stratification using the H2FPEF score may guide GDMT titration. For example, patients with H2FPEF scores ≥ 6 warrant SGLT2i initiation regardless of LVEF because of the demonstrated benefit on HFpEF [169]. Additionally, the potential anti-inflammatory effects of SGLT2i in HF patients may be relevant to MPNs pathophysiology [170]. SGLT2 inhibitors require cautious use because of their hematologic effects. Small cohort studies report consistent hematocrit elevation post-initiation, with thrombosis observed in PV, but potential anemia benefit in myelofibrosis. Close hematocrit monitoring is essential before and after SGLT2i initiation [160, 171].

Conversely, extreme thrombocytosis (> 1000 × 10⁹/L) mandates temporary aspirin interruption to avoid acquired von Willebrand syndrome [172]. Moreover, traditional CV risk factors such as hypertension, diabetes, and smoking remain clinically relevant (particularly in PV), despite their omission from certain revised thrombotic models [173]. In addition, AF adds a significant cardiovascular burden in MPNs, and is linked to an increased risk of HF hospitalization, cardiovascular death, and thrombotic events, underscoring the need for MPNs-adapted or hybrid scoring systems, as traditional scores such as the CHA₂DS₂-VASc score may underestimate the true risk [174].

When HF decompensation occurs during cytoreductive or JAK-inhibitor therapy (often precipitated by ruxolitinib-induced anemia, thrombocytopenia, fluid retention, or volume shifts), clinicians must weigh interrupting disease-modifying treatment against cardiotoxic contribution. Temporary dose reduction/interruption with rechallenge may be appropriate with clear temporal association and acceptable thrombotic risk [175, 176]. Conversely, when HF stems primarily from uncontrolled MPNs-related inflammation, hyperviscosity (leukocytosis/thrombocytosis), or severe splenomegaly, maintaining or even optimizing MPNs-directed therapy is crucial for long-term cardiovascular stability, as JAK inhibitors reduce thromboembolic events and hydroxyurea controls arterial thrombosis despite manageable hematologic toxicity [161, 177].

### Future directions. Hybrid risk stratification: practical algorithm

Patients with MPNs and HF represent a unique cardio-hematologic population where standard CV risk models underestimate disease burden. The H2FPEF score, validated in HFpEF, shows promise in MPNs, as higher scores correlate with increased rates of HF hospitalization. Hematologic factors such as leukocytosis and splenomegaly, indicate a direct contribution of the disease to cardiac dysfunction [164, 178].

**Fig. 2:**
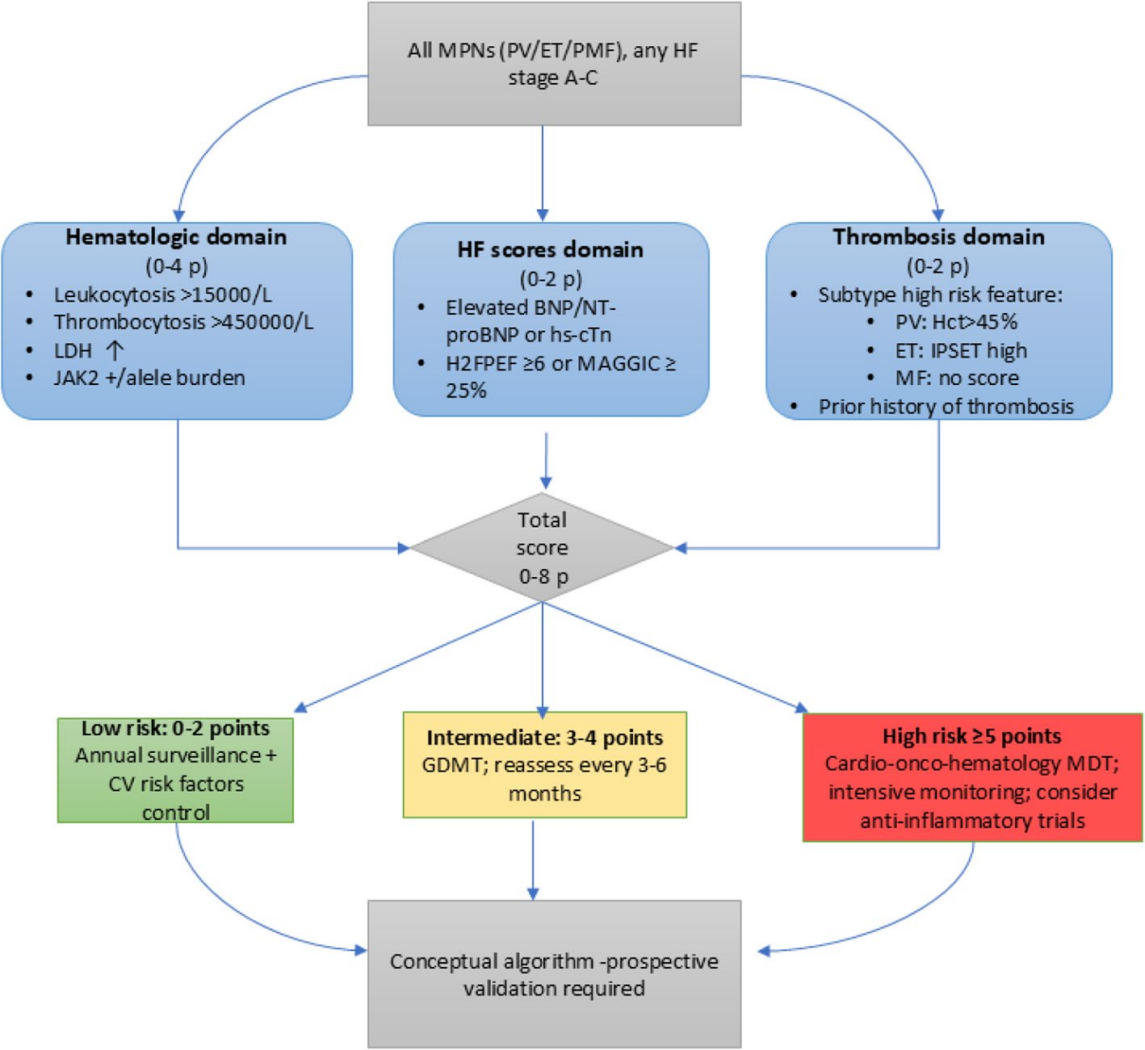
Proposed risk statification algorithm (0-8p). See abbreviations list

As no validated HF-MPNs risk model currently exists, we propose a pragmatic framework combining general HF prognostic scores (MAGGIC for mortality prediction or H2FPEF for HFpEF diagnosis/prognosis), along with MPNs-specific thrombotic scores, and hematologic parameters (leukocyte/platelet counts, JAK2 V617F presence/allele burden, LDH) [179–182]. The proposed algorithm (Fig. 2) outlines personalized management requiring prospective validation but aligns with ESC cardio-oncology principles of integrated risk assessment [163, 165].

This 0–8 point composite algorithm is designed to be applicable to all MPNs patients (ET/PV/MF) across AHA/ACC Stages A-C, irrespective of HF diagnosis, but it should currently be regarded as conceptual, pending prospective validation. Stage D (refractory end-stage HF) patients require specialized advanced therapies beyond this framework [12].

Scoring rationale: each independent risk factor contributes 1 point, reflecting established 2–3 × hazard ratios from the cardio-oncology/MPNs literature [8, 163, 173]. The simple additive model facilitates bedside use without software, generating actionable stratification (intensified surveillance, prevention, and therapy optimization) pending prospective validation. Unlike prior HFpEF frameworks, this algorithm explicitly incorporates Stages A and B, capturing asymptomatic MPNs patients with subclinical cardiac involvement for early preventive strategies.​

Moreover, prospective studies are needed to assess whether early GDMT or anti-inflammatory therapies (e.g., colchicine, canakinumab) reduce cardiovascular events in high-risk MPNs subsets [183]. Antifibrotic agents targeting lysyl oxidase-like 2 (LOXL2), which cross-links collagen and promotes stiffness, show promise for MPNs-associated HF across ACC/AHA Stages A–C: LOXL2 inhibition reduces interstitial fibrosis, improves diastolic function, and prevents chamber dilatation in stress-induced HF models, without affecting hypertrophy [184].

### Limitations

This is a narrative review, so the evidence is heterogeneous and subject to selection bias. Most data derive from separate HF or MPNs cohorts rather than patients with both conditions, and the proposed hybrid algorithm remains hypothesis-generating. External prospective validation will be required before clinical implementation.

Integration of artificial intelligence and machine learning (ML) with serial biomarkers, imaging, and hematologic data may enable more dynamic HF risk prediction in MPNs, but current ML models have not yet clearly outperformed traditional risk scores and still require careful validation and clinical integration; establishing multidisciplinary cardio-hematology clinics will be crucial to translate these tools into practice [185–187].

## Conclusions

This review highlights that no single biomarker or risk score adequately captures the bidirectional cardio-hematologic risk in MPNs-HF patients. Integrating established HF tools (NT-proBNP/troponins, H2FPEF/MAGGIC) with MPNs-specific parameters (JAK2 mutation/burden) within our proposed hybrid algorithm (Fig. 2) may facilitate more comprehensive risk stratification across ACC/AHA Stages A–C.

Prospective validation in dedicated cardio-oncology cohorts remains essential to refine this framework and establish MPNs-adapted HF guidelines. Multidisciplinary cardio-hemato-oncology teams, empowered by serial biomarker/imaging surveillance, represent the optimal pathway to mitigate HF progression, optimize GDMT alongside cytoreductive therapies, and improve survival in this vulnerable population.

## Data Availability

No datasets were generated or analysed during the current study.

## Abbreviations

3A3B: Age, albumin, anemia, BMI, BNP, BUN
ADHERE: Acute Decompensated Heart Failure National Registry
AF: Atrial fibrillation
ANC: Absolute neutrophil count
ARNI: Angiotensin receptor-neprilysin inhibitor
ASXL1: Additional Sex Combs-Like 1
BCN Bio-HF: Barcelona Bio-Heart Failure
BCOR/BCORL1: BCL6 Corepressor/BCL6 Corepressor Like 1
BMI: Body mass index
BNP: B-type natriuretic peptide
CALR: Calreticulin
CAR: C-reactive protein-to-albumin ratio
CEBPA: CCAAT/enhancer-binding protein alpha
CMR: Cardiac magnetic resonance
CRP: C-reactive protein
CV: Cardiovascular
CVRFs: Cardiovascular risk factors
DIPSS: Dynamic International Prognostic Scoring System
DNMT3A: DNA cytosine-5-methyltransferase 3 A
E/e: Early diastolic mitral inflow velocity to mitral annular early diastolic velocity ratio
EF: Ejection fraction
EHMRG: Emergency Heart Failure Mortality Risk Grade
ET: Essential thrombocythemia
EZH2: Enhancer of Zeste Homolog 2
Gal-3: Galectin-3
GDF-15: Growth differentiation factor-15
GDMT: Guideline-directed medical therapy
GLS: Global longitudinal strain
GM-CSF: Granulocyte–macrophage colony-stimulating factor
GWTG-HF: Get With The Guidelines-Heart Failure
Hb: Hemoglobin
HF: Heart failure
HFrEF: Heart failure with reduced ejection fraction
HFmrEF: Heart failure with mildly reduced ejection fraction
HFpEF: Heart failure with preserved ejection fraction
hs-cTnT: High-sensitivity cardiac troponin T
H2FPEF: Heart Failure with Preserved Ejection Fraction
IDH1/IDH2: Isocitrate dehydrogenase ½
IP-10: Interferon gamma-induced protein 10
IPSET: International Prognostic Score for Essential Thrombocythemia
JAK2: Janus kinase 2
LAVI: Left atrial volume index
LGE: Late gadolinium enhancement
LOXL2: Lysyl oxidase-like 2
LV: Left ventricle
LVEF: Left ventricular ejection fraction
LVMI: Left ventricular mass index
MAGGIC: Meta-Analysis Global Group in Chronic Heart Failure
MDT: Multidisciplinary team
MEDIA: M-mode Echocardiography-Derived Index for Assessment
MELD-XI: Model for End-Stage Liver Disease excluding INR
MFPS-PV: Multiple Factor Prognostic Score for PV
MIPSS: Mutation-Enhanced International Prognostic Scoring System
ML: Machine learning
MLR: Monocyte-to-lymphocyte ratio
MPL: Myeloproliferative leukemia virus oncogene
MPNs: Myeloproliferative neoplasms
mOS: Median overall survival
NLR: Neutrophil-to-lymphocyte ratio
NT-proBNP: N-terminal pro-B-type natriuretic peptide
NYHA: New York Heart Association
PALS: Left atrial reservoir strain
PASP: Pulmonary artery systolic pressure
PDGF: Platelet-derived growth factor
PH: Pulmonary hypertension
Plt: Platelets
PMF: Primary myelofibrosis
PV: Polycythemia vera
r-IPSET-T: Revised International Prognostic Score for Thrombosis in Essential Thrombocythemia
RUNX1: Runt-related transcription factor 1
SBP: Systolic blood pressure
SF3B1: Splicing factor 3b subunit 1
SGLT2i: Sodium-glucose cotransporter-2 inhibitor
SH2B3: SH2B adaptor protein 3
SHFM: Seattle Heart Failure Model
SII: Systemic immune-inflammation index
SRSF2: Serine and Arginine Rich Splicing Factor 2
sST2: Soluble Suppression of Tumorigenicity 2
TAPSE: Tricuspid annular plane systolic excursion
TNF-α: Tumor necrosis factor-alpha
U2AF1: U2 small nuclear RNA auxiliary factor 1
VEGF: Vascular endothelial growth factor
VTE: Venous thromboembolism
ULN: Upper limit of normal
WBC: White blood cell
